# Hypertension crises and management during radiofrequency ablation of adrenal pheochromocytoma: A case report

**DOI:** 10.1016/j.radcr.2024.06.024

**Published:** 2024-07-06

**Authors:** Mohadese Ahmadzade, Hamidreza Rouientan, Shahram Akhlaghpoor

**Affiliations:** Department of Interventional Radiology, Pardis Noor Medical Imaging and Cancer Center, Tehran, Iran

**Keywords:** Radiofrequency ablation, Pheochromocytoma, Hypertension crises, Interventional radiology

## Abstract

This case report describes the radiofrequency (RF) ablation of a pheochromocytoma in a 35-year-old female with multiple endocrine neoplasia (MEN) II syndrome, who previously underwent a right adrenalectomy and thyroidectomy. The patient presented with a new tumor in the left adrenal gland, detected via imaging, without evidence of metastasis. Opting against surgical adrenalectomy due to previous surgeries, she underwent RF ablation after preparatory alpha and beta blockader. During RF ablation, a hypertensive crisis occurred, managed effectively with nitroprusside sodium and supportive measures. Postprocedure recovery was uneventful, with normal metanephrine levels and imaging indicating successful ablation. This report highlights the feasibility and challenges of using RF ablation for adrenal pheochromocytoma, suggesting a potential shift towards less invasive management for select cases.

## Introduction

Over the past decade, minimally invasive technologies have been widely used to thermally ablate solid tumors within large organs by employing radiofrequency (RF) ablation, microwave thermal ablation, cryoablation, and high-intensity focused ultrasound. Thermal ablation has been applied primarily to the treatment of inoperable malignancies, primarily liver and kidney, as well as bone, lung, and breast cancers [1,2]. Despite the clinical focus of thermal ablation on malignancy, it offers potential for definitive treatment of benign endocrine tumors, particularly within the context of systemic endocrinopathy [3].

Bilateral functioning adrenal tumors are typically ineligible for resection due to the lack of feasible options for adrenal sparing surgery, along with the difficulty in identifying and localizing hyperfunctioning adrenal regions [4]. A precision application system for thermal ablation of the adrenal gland allows selective disruption of diseased tissue within one or both adrenal glands while preserving adjacent normal tissue unaffected – minimizing the risk of postprocedural adrenocortical insufficiency. Although some studies investigated the use of RF ablation to treat functioning adrenal adenoma such as primary aldosteronism and Cushing's syndrome, few reports have described the clinical utility of RF ablation in the treatment of pheochromocytoma [5].

In this case report, we describe the treatment of a left adrenal pheochromocytoma using RF ablation in a multiple endocrine neoplasia (MEN) II patient who had previously undergone a right adrenalectomy.

## Case presentation

A 35-year-old female, diagnosed with MEN II syndrome, who previously underwent thyroidectomy for medullary thyroid cancer and right adrenalectomy for pheochromocytoma, was referred with a newly diagnosed right adrenal pheochromocytoma.

**Fig. 1:**
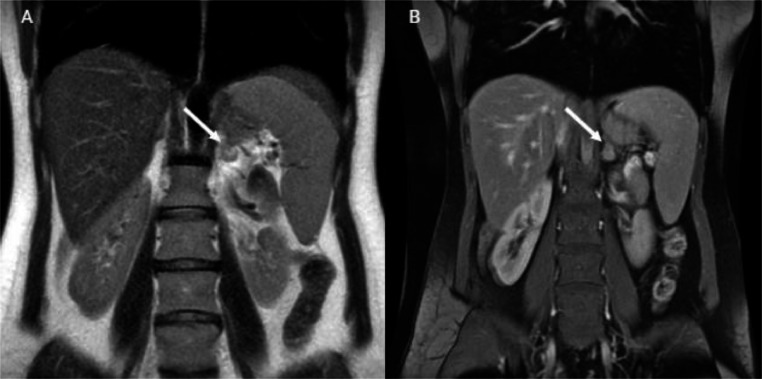
Coronal view of contrast-enhanced magnetic resonance imaging. (A) T2-weighted image revealing an iso-signal lesion in left adrenal gland (arrow). (B) T1-weighted image demonstrating a moderately enhanced lesion in the left adrenal gland (arrow).

An enhanced tumor was identified by magnetic resonance imaging in the left adrenal gland measuring 19 by 15 mm (Fig. 1). PET scans with ^68^Ga –DOTATATE revealed dense accumulation in the tumor, but no evidence of metastases elsewhere (Supplementary Material 1).

In this case, the patient was asymptomatic, with the tumor identified through follow-up imaging. The blood pressure measured 138/82 mmHg. The patient declined the surgical adrenalectomy and was thus referred for ablation as an alternative. Preparatory measures included administration of an Alpha blocker (phenoxybenzamine 10 mg twice daily) 10 days before, and a Beta blocker (propranolol 40 mg twice daily) 1 week prior to the procedure. On admission, blood pressure was 128/74 mm Hg with a heart rate of 66 beats/min. Alpha and Beta blockers were continued in oral dosages until the morning of the procedure. A normal blood test and laboratory results were obtained, except for an elevated level of urine metanephrine and normetanephrine.

**Fig. 2:**
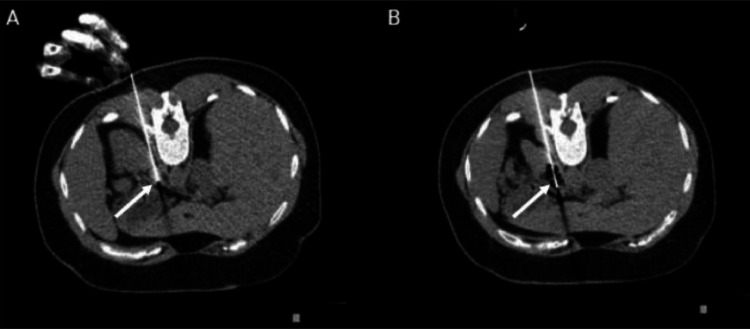
CT fluoroscopy-guided RF ablation. (A) Accurate needle placement under CT fluoroscopic guidance. (B) Conclusion of the ablation process is evidenced by gas accumulation around the needle's tip (arrow).

Under real-time CT fluoroscopy in prone position, a 17-G internally cooled RF electrodes (Medtronic Cool-tip RF Ablation System) with an exposed tip of 3 cm was placed within the tumor (Fig. 2). The RF electrode was connected to a generator and a switching controller (Cool-tip generator; Medtronic), which employed an impedance control algorithm to apply RF energy.

**Fig. 3:**
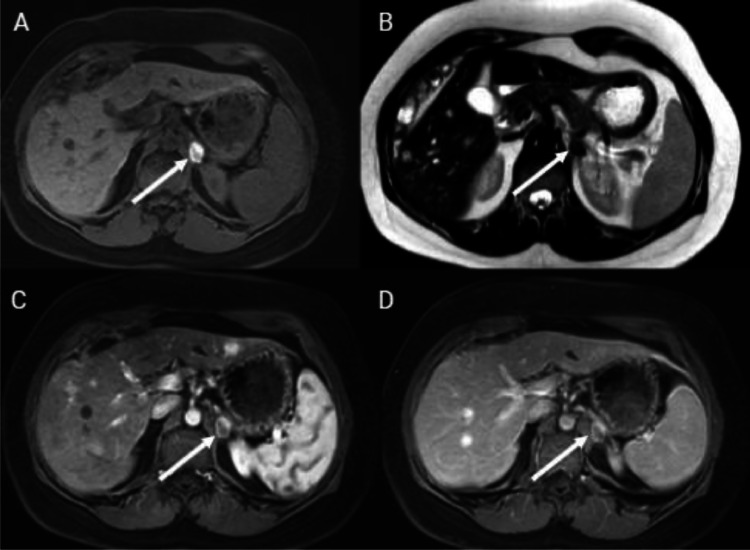
One month post-ablation axial contrast-enhanced magnetic resonance imaging. (A) The T1-fat saturated sequence reveals a high signal lesion consistent with post-ablation coagulative necrosis (arrow). (B) The lesion exhibits low signal intensity in the T2 non-fat saturated sequence (arrow). (C) There is minimal to no enhancement of the lesion in the arterial contrast-enhanced phase, and (D) in the delayed contrast-enhanced phase (arrow).

RF energy was applied using an 8-minute impedance control protocol. An arterial line was used to monitor blood pressure in real time via the radial artery. During RF ablation, a rapid rise in systolic blood pressure (210 mm Hg) was observed in relation to a rapid rise in heart rate (118 beat /min). RF ablation was suspended and nitroprusside sodium 1.8 μg/min was administered to control blood pressure. Immediately following the spike in blood pressure, the patient's blood pressure fell to 90/60 mm Hg. A combination of epinephrine and IV fluid was administered to increase her blood pressure and the RF ablation procedure was restarted, and the ablation process was continued. Gradually the patient's oxygen saturation dropped to 75%, pulmonary edema was diagnosed and Lasix 40 mg, aminophylline 30 mg/hour, and morphin 3 mg were prescribed for treatment. During this treatment, the RF ablation was performed and completed. The postablation course was uneventful, and the patient was admitted to the intensive care unit for further observation. The patient was administered a low dose of norepinephrine during her stay in the intensive care unit for twelve hours, and then the dose was tapered and discontinued. Blood pressure was stable without drug administration and the patient was discharged the following day. Two weeks after the procedure, plasma levels of free metanephrine were within normal ranges. One month after RF ablation procedure, magnetic resonance imaging was performed. Specifically, the T1-fat saturated non contrast sequence displayed a high signal intensity lesion that aligned with the expected post-ablation coagulative necrosis and post contrast study revealed no significant enhancement within the lesion (Fig. 3). During the follow-up period, the patient remained asymptomatic.

## Discussion

Pheochromocytoma is a rare tumor of the adrenal medulla or paraganglia, with an incidence of 1-5 cases per million [6]. Adrenalectomy is considered the gold standard of treatment, but the decision to proceed with surgery can be constrained by the presence of comorbid condition [7]. Thermal ablation may therefore represent a paradigm shift in definitive management for unilateral adrenal tumors, in addition to treating endocrinopathies caused by bilateral localized adrenal disease. With this percutaneous technique, adrenal tumors can be disrupted quickly and precisely while reducing patient discomfort, costs, and hospitalizations. Its indications extend to patients who are unwilling or unable to undergo adrenalectomy due to co-morbidities [8].

The most serious risk of adrenal ablation is the occurrence of hypertensive crisis with reported incidences ranging from 5.6% to 43.0%, resulting from medullary degranulation. This can lead to cardiomyopathy, pulmonary edema, or even circulatory collapse [9]. The lack of reports describing the RF ablation of pheochromocytoma may be attributed to the possibility of hypertensive crises. In order to keep monitoring the blood pressure, an arterial line should be secured due to the possibility of rapid hemodynamic changes. In the event of a hypertensive crisis, interventional radiologists should be prepared to turn off the radiofrequency generator immediately, since intravenous medications alone may not be sufficient to control the crisis.

According to the literature, biochemical outcomes following RF ablation and microwave ablation for endocrinopathy caused by aldosterone-producing adenoma or cortisol-secreting adenoma ranged from 75 to 100 percent for 1 ablation and 100% for repeated ablations. These results were found to be on par with those of unilateral adrenalectomy [5,10,11].

In conclusion, despite potential complications such as hypertensive crisis, with meticulous preparation and monitoring, RF ablation can achieve favorable outcomes. Further research and case accumulation are essential to validate the safety and efficacy of RF ablation in this context.

## Ethical approval

All procedures performed in studies involving human participants were in accordance with the ethical standards of the institutional and/or national research committee and with the 1964 Helsinki declaration and its later amendments or comparable ethical standards.

## Consent for publication

Consent for publication was obtained for every individual person's data included in the study.

## Patient consent

A written informed consent for publication of this case was obtained from the patient.

## Appendix Supplementary materials

Image, application 1
