# Supplementary material for: Hypertension crises and management during radiofrequency ablation of adrenal pheochromocytoma: A case report
Source: Radiol Case Rep. 2024 Jul 6;19(9):4012–6. doi: 10.1016/j.radcr.2024.06.024 (PMC11282923; doi:10.1016/j.radcr.2024.06.024)
Supplement: Supplementary file 1 [file mmc1.docx]

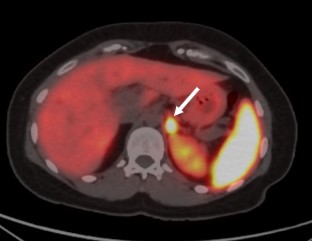


Supplementary Material 1: ^68^Ga –DOTATATE PET-CT Scan Image. The axial image demonstrating increased left adrenal uptake, indicated by the arrow, suggesting a somatostatin receptor-rich lesion compatible with a pheochromocytoma.
